# Presence and Persistence of Zika Virus RNA in Semen, United Kingdom, 2016

**DOI:** 10.3201/eid2304.161692

**Published:** 2017-04

**Authors:** Barry Atkinson, Fiona Thorburn, Christina Petridou, Daniel Bailey, Roger Hewson, Andrew J.H. Simpson, Timothy J.G. Brooks, Emma J. Aarons

**Affiliations:** Public Health England, Porton Down, UK (B. Atkinson, F. Thorburn, C. Petridou, D. Bailey, R. Hewson, A.J.H. Simpson, T.J.G. Brooks, E.J. Aarons);; West of Scotland Specialist Virology Centre, Glasgow, Scotland, UK (F. Thorburn);; Hampshire Hospitals National Health Service Foundation Trust, Basingstoke and Winchester, UK (C. Petridou).

**Keywords:** Zika, semen, sexual transmission, flavivirus, viruses, mosquitoborne, vector-borne infections, infectious diseases, United Kingdom, RNA, persistence, Zika virus

## Abstract

Zika virus RNA has been detected in semen collected several months after onset of symptoms of infection. Given the potential for sexual transmission of Zika virus and for serious fetal abnormalities resulting from infection during pregnancy, information regarding the persistence of Zika virus in semen is critical for advancing our understanding of potential risks. We tested serial semen samples from symptomatic male patients in the United Kingdom who had a diagnosis of imported Zika virus infection. Among the initial semen samples from 23 patients, Zika virus RNA was detected at high levels in 13 (56.5%) and was not detected in 9 (39.1%); detection was indeterminate in 1 sample (4.4%). After symptomatic infection, a substantial proportion of men have detectable Zika virus RNA at high copy numbers in semen during early convalescence, suggesting high risk for sexual transmission. Viral RNA clearance times are not consistent and can be prolonged.

Zika virus is an emerging flavivirus currently causing a major outbreak of human disease in the Americas, the Caribbean, and Western Pacific regions; 71 countries and territories have reported mosquitoborne transmission since 2015 (*1*). Human infection can be subclinical (*2*). Symptomatic infection usually causes a mild, self-limiting illness accompanied by rash, fever, arthralgia, and myalgia (*2*–*5*). However, international scientific consensus holds that Zika virus infection is a cause of 2 severe clinical sequelae recognized recently: Guillain-Barré syndrome and congenital neurologic abnormalities, including microcephaly (*6*). The risk for adverse obstetric outcome after maternal infection during pregnancy is currently unknown, but the risk for microcephaly attributable to Zika virus infection in the first trimester has been estimated to be 0.88%–13.2% (*7*).

Zika virus is primarily mosquitoborne, but sexual transmission has also been described (*8*–*14*). To date, 12 countries have reported nonvectorborne transmission (*1*), usually sexual transmission from men after symptomatic infection, although transmission after asymptomatic infection also has been described (*15*,*16*). Sexual transmission is a major concern for pregnant women and couples considering pregnancy because of the risk for adverse fetal sequelae. Information on Zika virus persistence in semen is required to inform public health guidance for the prevention of sexual transmission. With few exceptions (*17**,**18*), published data are mostly limited to individual case reports, and few publications report isolation of infectious Zika virus from semen. However, Zika virus RNA has been detected in semen up to 6 months after onset of symptoms (*19*,*20*). This potential for prolonged onward transmission warrants further investigation. We describe the analysis of serial semen samples from 23 symptomatic Zika virus–infected male travelers to determine the presence and persistence of Zika virus RNA.

## Methods

### Testing of Diagnostic Samples

We diagnosed possible imported Zika virus infection in patients by using a real-time reverse transcription PCR (rRT-PCR) assay based on a published method targeting the nonstructural protein 1 (NS1) gene (*21*) and a commercial serologic assay (EUROIMMUN AG, Lübeck, Germany). (We diagnosed infection in patient 1 by using a different rRT-PCR assay [*22*]). We performed viral RNA extraction on the EZ1 platform by using the EZ1 Virus Mini Kit with Buffer AVL (QIAGEN, Valencia, CA, USA) inactivated samples. We also tested all patients for other pathogens, such as chikungunya and dengue viruses, by using published molecular and commercial serologic assays. The preferred sample type was serum or plasma, but published testing guidance specifically called for urine samples from pregnant women and male partners of pregnant women.

### Testing of Semen Samples

We offered a Zika virus RNA semen testing service to adult male patients with diagnosed Zika virus infection. If Zika virus RNA was detected, we offered testing of serial samples. We advised that samples be collected in sterile pots and transported at ambient temperature. We analyzed the samples by using the RNA extraction and rRT-PCR testing procedures described here, without modification.

## Results

Since December 2013, nearly 2,500 Zika virus diagnostic molecular detection tests have been performed on travelers returning to the United Kingdom with possible Zika virus infection. As of October 5, 2016, a total of 116 persons, 55 of whom were males, had detectable Zika virus RNA in serum, plasma, or urine (*23*). Sixteen male patients (29.1%) submitted >1 semen sample (patients 1–16). In addition, 7 patients (patients 17–23) submitted semen samples after serologic diagnoses (i.e., detection of Zika virus IgM and IgG in serum but without detection of Zika virus RNA).

All patients had recently traveled from the Americas or Caribbean, except patient 1, who acquired Zika virus infection in the South Pacific in 2014. All patients reported a self-limiting, mild illness with fever and/or rash consistent with Zika virus infection (Technical Appendix Tables 1 and 2). None reported hematospermia.

We detected Zika virus RNA in >1 semen sample from 10 (62.5%) of the 16 patients with rRT-PCR–diagnosed infection and from 2 (28.6%) of the 7 patients with serologically diagnosed infection. For the 10 patients who had a positive initial blood or urine sample and a subsequent positive semen sample, the median cycle threshold (C_t_) value was significantly lower for semen samples (C_t_ 27.3) than for acute-phase diagnostic serum, plasma, or urine samples (C_t_ 34.1; p = 0.01 by sign test). 

Further semen samples were provided by 8 of the 12 Zika virus–positive patients (Figure). A series of samples sufficient to demonstrate seminal clearance of Zika virus RNA was available for 4 patients (*2*,*5*,*9*,*10*). Patient 5 demonstrated the longest time to clearance (from day 92 to day 131). For other patients from whom additional positive semen samples were received, C_t_ values increased consistently over time (i.e., genome copy number fell). The day 167 semen sample of patient 8 repeatedly demonstrated a subthreshold curve in the rRT-PCR test, suggesting evidence of Zika virus RNA at the assay detection limit. This patient was taking immunosuppressive drug therapy when infected (disease-modifying antirheumatic drugs [discontinued after his diagnoses with Zika virus infection] and oral steroids). No other patients were known to have immunosuppression.

**Figure:**
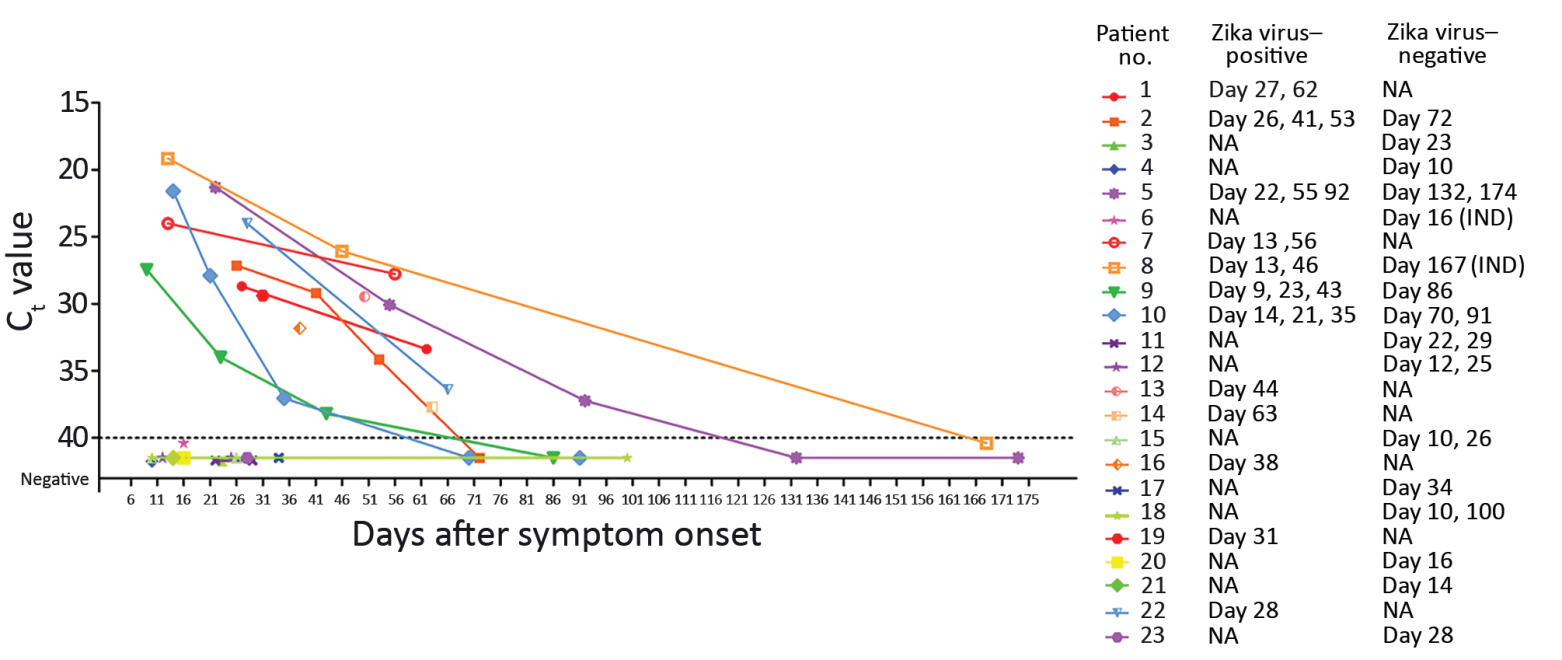
Cycle threshold (C_t_) values of semen samples analyzed to determine presence and persistence of Zika virus RNA in semen, United Kingdom, 2016. All references to days indicate days after onset of symptoms. The dotted line represents the limit of detection for the assay. The lines represent implied decay rates based on longitudinal data; however, they should not be used to infer exact data between analyzed samples. In particular, the point at which viral RNA became undetected cannot be accurately inferred from the curves for patients 5, 8, 9, and 10. Patient 6 had a previous vasectomy, and patient 8 was receiving immunosuppressive drug therapy at the time of sampling. IND, indeterminate; NA, not available.

Zika virus RNA was undetectable in semen samples from 5 of the 16 patients with rRT-PCR–diagnosed infection (31.3%); results were indeterminate (subthreshold) for 1 patient (6.3%). All 6 patients subsequently seroconverted for Zika virus IgG (data not shown). The indeterminate result occurred in the only patient who had undergone a vasectomy (day 16 sample). Zika virus RNA was also undetectable in the semen of 5 (71.4%) of the 7 patients with serologically diagnosed infection. Although Zika virus infection was not definitively confirmed by molecular testing in any of these 5 patients, laboratory tests excluded recent chikungunya or dengue infection (Technical Appendix Table 2).

Of the 15 patients with rRT-PCR–confirmed infection in whom previous dengue serostatus could be assessed at the time of their initial Zika virus diagnosis (because no potentially cross-reactive Zika virus IgG was detectable at that time), only 3 had serologic evidence of previous dengue virus infection. Two of these patients (patients 3 and 6) did not have Zika virus RNA in subsequent semen samples (Technical Appendix Table 1).

We attempted isolation of viable virus from all initial positive semen samples, with 1 successful isolation (day 13 sample from patient 7, C_t_ 24). We attempted virus isolation in Vero and C6/36 cells; we observed propagation only in C6/36 cells for the original sample, although C6/36 infected supernatant was capable of infecting Vero cells and produced a cytopathic effect. We deposited the complete genome for this isolate in GenBank (accession no. KX673530) (*24*).

## Discussion

Although persistence of Zika virus in serum and plasma appears to be transient, our data show that Zika virus RNA is frequently detectable in semen for several weeks to months after recovery from symptomatic infection. Zika virus RNA persistence in semen is not consistent; 10 (43.5%) of the 23 patients we tested lacked detectable Zika virus RNA in semen on the first sample (all but 1 tested within 28 days after symptom onset). Our detection of Zika virus RNA in early semen samples from 12 (52.2%) of 23 infected patients is similar to the detection of viral RNA in 50% of case-patients in small-number Belgian (*17*) and French (*18*) series.

We observed no obvious differences in terms of age, travel history, or symptoms between the patients with and without Zika virus RNA in our series, although detailed clinical information about ethnic origin, symptom severity or duration, and laboratory parameters, such as platelet count or C-reactive protein, were not collected. Sample degradation probably could not explain the negative results because the time from sample collection to testing was shorter on average for negative samples than for samples yielding positive results (data not shown).

One patient with an indeterminate rRT-PCR result for his initial (day 16) semen sample was reported to have had a vasectomy; ongoing viral replication might have been occurring in the urogenital tract. A recent publication reported Zika virus RNA in the ejaculate of a vasectomized man 96 days after onset of symptoms in addition to infectious virus cultured at 69 days (*25*), suggesting that previous vasectomy does not necessarily preclude the possibility of viral RNA or infectious virions in the semen.

It has been hypothesized that previous dengue infection might drive greater Zika virus replication through the mechanism of antibody-dependent enhancement; this process has been observed in vitro (*26*,*27*). However, our data indicate that this process cannot be invoked as a potential sole explanation for seeding of infection in the urogenital tract; among the 9 patients with Zika virus RNA in their semen for whom dengue serostatus at the time of Zika virus infection could be determined, only 1 (patient 10) had serologic evidence of previous dengue virus infection. Our data are insufficient to allow speculation as to whether an altered immune response associated with previous dengue infection might protect against viral replication in the male urogenital tract.

Among the 12 patients in whom Zika virus RNA was detected in semen, all samples obtained within 28 days of symptom onset had C_t_ values <30, indicative of high genome copy number. This observation is concordant with other reports (*9*,*12*,*17*,*19*,*28*–*31*) and suggests that the risk for sexual transmission is particularly high in the first few weeks after infection.

The specimens analyzed in this report were provided at the discretion of the patients, and the data are subject to limitations. Monitoring was inconsistent, and patients were lost to follow-up. Clearance of Zika virus RNA from semen was demonstrated for only 4 patients. In all other patients from whom serial Zika virus RNA–positive semen samples were received, the C_t_ value increased over time, consistent with a fall in genome copy number, as has been observed previously (*12*,*17*,*30*,*32*). The patient with an indeterminate (subthreshold) result at 167 days (patient 8) was receiving immunosuppressive therapy. Because Zika virus is the only known arbovirus with evidence for human sexual transmission, we cannot compare the persistence of Zika virus RNA or infectious virions in semen to similar viruses. Although the presence of Zika virus in semen is presumed to be attributable to viral replication at an immunoprivileged site within the urogenital tract, the cell type in which replication occurs is not currently known. In a mouse model, viral RNA was detected in the testes, in spermatogonia, primary spermatocytes, and Sertoli cells (*33*)

Detection of Zika virus RNA in semen samples does not necessarily indicate the presence of infectious virus. We attempted in vitro isolation from all positive samples; only 1 sample (day 13 sample from patient 7, C_t_ 24) yielded replicating virus. Isolation from samples with similar C_t_ values was unsuccessful, highlighting the inconsistency for this technique at present. The inability to isolate virus cannot be taken to indicate the absence of infectious virus until more is known about the intricacies of virus isolation from semen. Although we have been unable to isolate virus in tissue culture consistently from semen samples, it is striking that all recognized transmission events to date have occurred relatively soon after infection in the male index patient (*8*–*13*,*34*). The longest published interval for sexual transmission after symptom onset is 32–41 days (*14*). Even if methodology for reliably isolating Zika virus from semen samples is established, it will be difficult to determine whether semen samples with Zika virus RNA detected at lower copy numbers contain infectious virions.

The current World Health Organization (WHO) guidelines advise that male and female travelers should adopt safer sex practices or consider abstinence to reduce the risk for sexual transmission for 6 months after leaving a country with ongoing Zika virus transmission (*35*). This recommendation was a conservative estimate for symptomatic men given the maximum reported persistence of Zika virus RNA in semen (62 days) at the time the WHO guidelines were issued (May 2016). Our data, together with other recent case reports (*12*,*18*–*20*,*25*,*32*,*36*), show that Zika virus RNA is sometimes detectable beyond 62 days, with the maximum period of RNA detection observed to date being approximately 6 months (*20*). However, our understanding of the dynamics for virus or viral RNA clearance are still nascent, and further data are required to assess the validity of current recommendations.

Currently, we lack knowledge of viral persistence in semen. More data, especially from larger systematic studies, are urgently needed to support evidence-based policies to prevent sexual transmission of Zika virus. In the meantime, testing of semen from symptomatic men >8 weeks after leaving a Zika-affected area, as suggested by WHO (*35*), might be valuable for assessing individual risk and for contributing to global data.

Technical Appendix Characteristics of patients with real-time reverse transcription PCR–confirmed and serologically diagnosed Zika virus infection who were evaluated to determine presence and persistence of Zika virus RNA in semen, United Kingdom, 2016.

## References

[1] World Health Organization. Zika situation report, 29 September 2016 [cited 2016 Oct 9]. http://www.who.int/emergencies/zika-virus/situation-report/29-september-2016/en

[2] Duffy MR, Chen T-H, Hancock WT, Powers AM, Kool JL, Lanciotti RS, et al. Zika virus outbreak on Yap Island, Federated States of Micronesia. N Engl J Med. 2009;360:2536–43. 10.1056/NEJMoa0805715 19516034

[3] Brasil P, Calvet GA, Siqueira AM, Wakimoto M, de Sequeira PC, Nobre A, et al. Zika virus outbreak in Rio de Janeiro, Brazil: clinical characterization, epidemiological and virological aspects. PLoS Negl Trop Dis. 2016;10:e0004636. 10.1371/journal.pntd.0004636 27070912 PMC4829157

[4] Cerbino-Neto J, Mesquita EC, Souza TML, Parreira V, Wittlin BB, Durovni B, et al. Clinical manifestations of Zika virus infection, Rio de Janeiro, Brazil, 2015. Emerg Infect Dis. 2016;22:1318–20. 10.3201/eid2207.160375 27070847 PMC4918188

[5] Thomas DL, Sharp TM, Torres J, Armstrong PA, Munoz-Jordan J, Ryff KR, et al. Local Transmission of Zika Virus—Puerto Rico, November 23, 2015-January 28, 2016. MMWR Morb Mortal Wkly Rep. 2016;65:154–8. 10.15585/mmwr.mm6506e2 26890470

[6] World Health Organization. Zika causality statement [cited 2016 Oct 9]. http://www.who.int/emergencies/zika-virus/causality/en

[7] Johansson MA, Mier-y-Teran-Romero L, Reefhuis J, Gilboa SM, Hills SL. Zika and the risk of microcephaly. N Engl J Med. 2016;375:1–4. 10.1056/NEJMp1605367 27222919 PMC4945401

[8] Deckard DT, Chung WM, Brooks JT, Smith JC, Woldai S, Hennessey M, et al. Male-to-male sexual transmission of Zika virus—Texas, January 2016. MMWR Morb Mortal Wkly Rep. 2016;65:372–4. 10.15585/mmwr.mm6514a3 27078057

[9] D’Ortenzio E, Matheron S, Yazdanpanah Y, de Lamballerie X, Hubert B, Piorkowski G, et al. Evidence of sexual transmission of Zika virus. N Engl J Med. 2016;374:2195–8. 10.1056/NEJMc1604449 27074370

[10] Foy BD, Kobylinski KC, Chilson Foy JL, Blitvich BJ, Travassos da Rosa A, Haddow AD, et al. Probable non-vector-borne transmission of Zika virus, Colorado, USA. Emerg Infect Dis. 2011;17:880–2. 10.3201/eid1705.101939 21529401 PMC3321795

[11] Frank C, Cadar D, Schlaphof A, Neddersen N, Günther S, Schmidt-Chanasit J, et al. Sexual transmission of Zika virus in Germany, April 2016. Euro Surveill. 2016;21:30252. 10.2807/1560-7917.ES.2016.21.23.30252 27311329

[12] Harrower J, Kiedrzynski T, Baker S, Upton A, Rahnama F, Sherwood J, et al. Sexual transmission of Zika virus and persistence in semen, New Zealand, 2016. Emerg Infect Dis. 2016;22:1855–7 . 10.3201/eid2210.160951 27454745 PMC5038405

[13] Hills SL, Russell K, Hennessey M, Williams C, Oster AM, Fischer M, et al. Transmission of Zika virus through sexual contact with travelers to areas of ongoing transmission—continental United States, 2016. MMWR Morb Mortal Wkly Rep. 2016;65:215–6. 10.15585/mmwr.mm6508e2 26937739

[14] Turmel JM, Abgueguen P, Hubert B, Vandamme YM, Maquart M, Le Guillou-Guillemette H, et al. Late sexual transmission of Zika virus related to persistence in the semen. Lancet. 2016;387:2501. 10.1016/S0140-6736(16)30775-9 27287833

[15] Brooks RB, Carlos MP, Myers RA, White MG, Bobo-Lenoci T, Aplan D, et al. Likely sexual transmission of Zika virus from a man with no symptoms of infection—Maryland, 2016. MMWR Morb Mortal Wkly Rep. 2016;65:915–6. 10.15585/mmwr.mm6534e2 27585037

[16] Fréour T, Mirallié S, Hubert B, Splingart C, Barrière P, Maquart M, et al. Sexual transmission of Zika virus in an entirely asymptomatic couple returning from a Zika epidemic area, France, April 2016. Euro Surveill. 2016;21:30254. 10.2807/1560-7917.ES.2016.21.23.30254 27311680

[17] Huits R, De Smet B, Ariën K, Van Esbroeck M, de Jong B, Bottieau E, et al. Kinetics of Zika virus persistence in semen. Bull World Health Organ Zika Open. 2016 Jul 6.

[18] Mansuy JM, Suberbielle E, Chapuy-Regaud S, Mengelle C, Bujan L, Marchou B, et al. Zika virus in semen and spermatozoa. Lancet Infect Dis. 2016;16:1106–7. 10.1016/S1473-3099(16)30336-X 27676340

[19] Barzon L, Pacenti M, Berto A, Sinigaglia A, Franchin E, Lavezzo E, et al. Isolation of infectious Zika virus from saliva and prolonged viral RNA shedding in a traveller returning from the Dominican Republic to Italy, January 2016. Euro Surveill. 2016;21. 10.2807/1560-7917.ES.2016.21.10.30159 26987769

[20] Nicastri E, Castilletti C, Liuzzi G, Iannetta M, Capobianchi MR, Ippolito G. Persistent detection of Zika virus RNA in semen for six months after symptom onset in a traveller returning from Haiti to Italy, February 2016. Euro Surveill. 2016;21:30314. 10.2807/1560-7917.ES.2016.21.32.30314 27541989 PMC4998502

[21] Pyke AT, Daly MT, Cameron JN, Moore PR, Taylor CT, Hewitson GR, et al. Imported Zika virus infection from the Cook Islands into Australia, 2014. PLoS Curr 2014;6: pii: ecurrents.outbreaks.4635a54dbffba2156fb2fd76dc49f65e. https://dx.doi.org/10.1371%2Fcurrents.outbreaks.4635a54dbffba2156fb2fd76dc49f65e 10.1371/currents.outbreaks.4635a54dbffba2156fb2fd76dc49f65e PMC4055592 24944843

[22] Lanciotti RS, Kosoy OL, Laven JJ, Velez JO, Lambert AJ, Johnson AJ, et al. Genetic and serologic properties of Zika virus associated with an epidemic, Yap State, Micronesia, 2007. Emerg Infect Dis. 2008;14:1232–9. 10.3201/eid1408.080287 18680646 PMC2600394

[23] Public Health England. Zika virus: epidemiology and cases diagnosed in the UK [cited 2016 Oct 9]. https://www.gov.uk/government/publications/zika-virus-epidemiology-and-cases-diagnosed-in-the-uk/zika-virus-epidemiology-and-cases-diagnosed-in-the-uk

[24] Atkinson B, Graham V, Miles RW, Lewandowski K, Dowall SD, Pullan ST, et al. Complete genome sequence of Zika virus isolated from semen. Genome Announc. 2016;4:e01116–16. 10.1128/genomeA.01116-16 27738033 PMC5064106

[25] Arsuaga M, Bujalance SG, Díaz-Menéndez M, Vázquez A, Arribas JR. Probable sexual transmission of Zika virus from a vasectomised man. Lancet Infect Dis. 2016;16:1107. 10.1016/S1473-3099(16)30320-6 27676342

[26] Dejnirattisai W, Supasa P, Wongwiwat W, Rouvinski A, Barba-Spaeth G, Duangchinda T, et al. Dengue virus sero-cross-reactivity drives antibody-dependent enhancement of infection with zika virus. Nat Immunol. 2016;17:1102–8. 10.1038/ni.3515 27339099 PMC4994874

[27] Stettler K, Beltramello M, Espinosa DA, Graham V, Cassotta A, Bianchi S, et al. Specificity, cross-reactivity, and function of antibodies elicited by Zika virus infection. Science. 2016;353:823–6. 10.1126/science.aaf8505 27417494

[28] Mansuy JM, Dutertre M, Mengelle C, Fourcade C, Marchou B, Delobel P, et al. Zika virus: high infectious viral load in semen, a new sexually transmitted pathogen? Lancet Infect Dis. 2016;16:405. 10.1016/S1473-3099(16)00138-9 26949027

[29] Musso D, Roche C, Robin E, Nhan T, Teissier A, Cao-Lormeau V-M. Potential sexual transmission of Zika virus. Emerg Infect Dis. 2015;21:359–61. 10.3201/eid2102.141363 25625872 PMC4313657

[30] Reusken C, Pas S. GeurtsvanKessel C, Mögling R, van Kampen J, Langerak T, et al. Longitudinal follow-up of Zika virus RNA in semen of a traveller returning from Barbados to the Netherlands with Zika virus disease, March 2016. Euro Surveill. 2016;21:30251. 10.2807/1560-7917.ES.2016.21.23.30251 27313200

[31] Jang H-C, Park WB, Kim UJ, Chun JY, Choi S-J, Choe PG, et al. First imported case of Zika virus infection into Korea. J Korean Med Sci. 2016;31:1173–7. 10.3346/jkms.2016.31.7.1173 27366020 PMC4901014

[32] Matheron S, d’Ortenzio E, Leparc-Goffart I, Hubert B, de Lamballerie X, Yazdanpanah Y. Long-lasting persistence of Zika virus in semen. Clin Infect Dis. 2016;63:1264. 10.1093/cid/ciw509 27470244

[33] Govero J, Esakky P, Scheaffer SM, Fernandez E, Drury A, Platt DJ, et al. Zika virus infection damages the testes in mice. Nature. 2016. 10.1038/nature20556 PMC5432198 27798603

[34] Venturi G, Zammarchi L, Fortuna C, Remoli ME, Benedetti E, Fiorentini C, et al. An autochthonous case of Zika due to possible sexual transmission, Florence, Italy, 2014. Euro Surveill. 2016;21:30148. 10.2807/1560-7917.ES.2016.21.8.30148 26939607

[35] World Health Organization. Prevention of sexual transmission of Zika virus. Interim guidance [cited 2016 Oct 9]. http://www.who.int/csr/resources/publications/zika/sexual-transmission-prevention/en

[36] Mansuy JM, Pasquier C, Daudin M, Chapuy-Regaud S, Moinard N, Chevreau C, et al. Zika virus in semen of a patient returning from a non-epidemic area. Lancet Infect Dis. 2016;16:894–5. 10.1016/S1473-3099(16)30153-0 27477981

