# Correction: Vol. 23, No. 4

**DOI:** 10.3201/eid2307.C12307

**Published:** 2017-07

**Keywords:** errata, erratum, correction

The number of semen samples with high Zika virus levels was incorrectly stated in the abstract of Presence and Persistence of Zika Virus RNA in Semen, United Kingdom, 2016 (B. Atkinson et al.). Zika virus RNA was detected at high levels in 12 (52.2%) samples and was not detected in 10 (43.5%) samples from 43 patients. The article has been corrected online (https://wwwnc.cdc.gov/eid/article/23/4/16-1692_article).

