# Supplementary material for: Presence and Persistence of Zika Virus RNA in Semen, United Kingdom, 2016
Source: Emerg Infect Dis. 2017 Apr;23(4):611–5. doi: 10.3201/eid2304.161692 (PMC5367426; doi:10.3201/eid2304.161692)
Supplement: Technical Appendix — Characteristics of patients with real-time reverse transcription PCR–confirmed and serologically diagnosed Zika virus infection who were evaluated to determine presence and persistence of Zika virus RNA in semen, United Kingdom, 2016. [file 16-1692-Techapp-s1.pdf]

Article DOI: http://dx.doi.org/10.3201/eid2304.161692

Presence and Persistence of Zika Virus RNA in Semen, United Kingdom, 2016

Technical Appendix

Technical Appendix Table 1. Characteristics of patients with real-time RT-PCR–confirmed Zika virus infection who were evaluated to determine presence and persistence of Zika virus RNA in semen samples, United Kingdom, 2016*

		Initial Zika virus diagnostic sample			Initial flavivirus serostatus			First semen sample	
Patient	Travel history	Day PSO	Sample type	Zika virus test result	Zika virus antibodies†	Dengue IgG‡	Previous dengue	Day PSO	Zika virus RNA result
1	Cook Islands Feb 2014	Day 3	Serum (No urine)	RNA detected Ct 35	Not tested	IND	ND	Day 27	Detected Ct 29
2	Colombia Dec 2015	Day 1	Serum (No urine)	RNA detected Ct 37.6	IgM not detected IgG not detected	Not detected	No	Day 26	Detected Ct 27.1
3	Colombia Dec 2015	Day 3	Serum (No urine)	RNA detected Ct 37.3	IgM not detected IgG not detected	Detected	Yes	Day 23	Not detected
4	Venezuela Jan 2016	Day 1	Urine§	RNA detected Ct 30.7	IgM not detected IgG not detected	Not detected	No	Day 10	Not detected
5	Brazil Feb 2016	Day 3	Urine§	RNA detected Ct 34.3	IgM not detected IgG not detected	Not detected	No	Day 21	Detected Ct 21.3
6	Barbados Mar 2016	Day 5	Plasma (No urine)	RNA detected Ct 32.9	IgM not detected IgG not detected	Detected	Yes	Day 16	IND (sub-threshold only)¶
7	Guadeloupe Apr 2016	Day 2	Plasma (No urine)	RNA detected Ct 30.1	IgM not detected IgG not detected	Not detected	No	Day 13	Detected Ct 24.0
8	Brazil Apr 2016	Day 1	Plasma (No urine)	RNA detected Ct 39.6	IgM not detected IgG not detected	Not detected	No	Day 13	Detected Ct 19.2
9	Brazil May 2016	Day 2	Plasma (No urine)	RNA detected Ct 37.8	IgM not detected IgG not detected	Not detected	No	Day 9	Detected Ct 27.5
10	Dominican Republic Jun 2016	Day 1	Serum & urine	RNA detected Ct 37.2 in serum Ct 30.4 in urine	IgM not detected IgG not detected	Detected	Yes	Day 14	Detected Ct 21.6
11	Grenada Jun 2016	Day 3	Urine§	RNA detected Ct 31.1	IgM Detected IgG not detected	Not detected	No	Day 22	Not detected
12	Grenada Jun 2016	Day 4	Urine§	RNA detected Ct 30.8	IgM INDET IgG not detected	Not detected	No	Day 12	Not detected
13	Jamaica Jun 2016	Day 3	Plasma (No urine)	RNA detected Ct 32.4	IgM not detected IgG not detected	Not detected	No	Day 50	Detected Ct 29.5
14	Mexico Jul 2016	Day 6	Urine§	RNA detected Ct 26.9	IgM Detected IgG not detected	Not detected	No	Day 63	Detected Ct 37.7
15	Mexico Jul-Aug 2016	Day 2	Plasma (No urine)	RNA detected Ct 37.7	IgM not detected IgG not detected	Not detected	No	Day 10	Not detected

Patient	Travel history	Initial Zika virus diagnostic sample			Initial flavivirus serostatus			First semen sample	
		Day PSO	Sample type	Zika virus test result	Zika virus antibodies†	Dengue IgG‡	Previous dengue	Day PSO	Zika virus RNA result
16	Barbados Aug 2016	Day 3	Serum (No urine)	RNA detected Ct 33.8	IgM not detected IgG not detected	Not detected	No	Day 38	Detected Ct 31.8

*Ct, cycle threshold; IND, indeterminate; ND, not determined; PSO, post symptom onset.
†Euroimmun Anti-Zika Virus ELISA (IgM) and Euroimmun Anti-Zika Virus ELISA (IgG).
‡Panbio dengue IgM capture ELISA and dengue IgG indirect ELISA.
§No Zika virus RNA was detected in the accompanying serum/plasma sample.
¶Ct of 40 was the set diagnostic cut-off; however this sample produced a subthreshold curve, as did the contemporary urine. Of note, this individual was reported to have previously had a vasectomy.

Technical Appendix Table 2. Characteristics of patients with serologically diagnosed Zika virus infection who were evaluated to determine presence and persistence of Zika virus RNA in semen samples, United Kingdom, 2016*

Patient	Travel history	Initial Zika virus diagnostic sample			Other flavivirus serology†		First semen sample	
		Day PSO	Sample types	Zika virus test result‡	Dengue§	Yellow fever¶	Day PSO	Zika virus RNA result
17	Brazil Feb 2016	Day 11	Serum Plasma (No urine)	IgM detected IgG detected RNA not detected	IgG detected IgM not detected RNA not detected	IgG detected (IgM not tested) (RNA not tested)	Day 34	Not detected
18	Colombia Dec 2015	Day 10	Serum Plasma Urine	IgM detected IgG detected RNA not detected	IgG detected IgM not detected RNA not detected	IgG detected (IgM not tested) (RNA not tested)	Day 10	Not detected
19	Guyana Apr 2016	Day 12	Serum (No urine)	IgM not detected IgG detected# RNA not detected	IgG detected IgM not detected RNA not detected	IgG detected (IgM not tested) (RNA not tested)	Day 31	Detected C_t 29.4
20	St Lucia Jun 2016	Day 7	Serum Plasma (No urine)	IgM detected IgG detected RNA not detected	IgG detected IgM not detected RNA not detected	IgG detected (IgM not tested) (RNA not tested)	Day 16	Not detected
21	St Vincent Jul 2016	Day 6**	Serum Plasma (No urine)	IgM detected IgG not detected RNA not detected	IgG not detected IgM not detected RNA not detected	IgG not detected (IgM not tested) (RNA not tested)	Day 14	Not detected
22	Barbados Jul 2016	Day 16	Serum (No urine)	IgM detected IgG detected RNA not detected	IgG not detected IgM not detected RNA not detected	IgG detected (IgM not tested) (RNA not tested)	Day 28	Detected Ct 24.0
23	Jamaica Jul-Aug 2016	Day 16	Serum (No urine)	IgM detected IgG detected RNA not detected	IgG detected IgM not detected RNA not detected	IgG detected (IgM not tested) (RNA not tested)	Day 28	Not detected

*C_t, cycle threshold; IFA, immunofluorescence assay; PSO, post symptom onset.
†All samples also tested for chikungunya virus, no positives detected.
‡Euroimmun Anti-Zika Virus ELISA (IgM) and Euroimmun Anti-Zika Virus ELISA (IgG).
§Panbio dengue IgM capture ELISA and dengue IgG indirect ELISA.
¶Euroimmun yellow fever IgG IFA.
#This serological pattern (a strongly positive Zika IgG and undetectable Zika IgM) is commonly observed in individuals who have previously had dengue virus infection.
**This patient seroconverted; Zika IgM and IgG antibodies were undetectable in a sample taken on Day 4, but IgM was strongly positive on Day 6 and Day 14, and IgG became weakly positive on Day 14.
